# CT-Based Adaptive Short Course Radiotherapy for Rectal Cancer: A Case Report

**DOI:** 10.7759/cureus.69264

**Published:** 2024-09-12

**Authors:** Jonathan Henning, Sylvia Choo, Pranit Singh, Gage Redler, Russell F Palm

**Affiliations:** 1 Radiation Oncology, University of South Florida Morsani College of Medicine, Tampa, USA; 2 Radiation Oncology, Moffitt Cancer Center, Tampa, USA; 3 Radiation Oncology, The Ohio State University Wexner Medical Center, Columbus, USA

**Keywords:** adaptive radiation, medical physics, metastatic rectal cancer, radiation therapy technology, rectal cancer, short course radiation

## Abstract

Rectal cancer is a highly prevalent malignancy with an increasing number of treatment strategies and sequencing. Short-course radiotherapy (SCRT) combined with chemotherapy has shown to be a feasible treatment option for non-metastatic disease within the total neoadjuvant therapy paradigm. In contrast to conventionally fractionated chemoradiation, SCRT carries less logistical burden and reduces time off systemic therapy, which is particularly beneficial for patients with metastatic disease. We present a case of a 42-year-old male with metastatic rectal cancer who received SCRT using CT-based adaptive radiation, delivering 25 Gy to the pelvis with a simultaneous integrated boost of 30 Gy to the rectal tumor over five treatments. CT-based adaptive radiation was effective and well-tolerated and offers a pathway towards isotoxic radiotherapy dose escalation for patients considered to be inoperable. This case demonstrates the integration of adaptive SCRT as a promising strategy for rectal cancer.

## Introduction

Colorectal cancer is the third most commonly diagnosed cancer worldwide, with common presenting symptoms including changes in bowel habits, hematochezia, tenesmus, abdominal discomfort, and weight loss [1]. Common sites of rectal cancer metastasis include the liver, lungs, and, less often, the peritoneum [2]. The treatment strategies for rectal cancer are rapidly evolving, with modern recommendations to incorporate pelvic MRI imaging at initial staging [3], which facilitates patient stratification towards optimal treatment sequencing to balance the risk of metastatic disease with the patient’s oncologic and functional outcome [4,5].

From a radiation oncology perspective, the use of short-course radiotherapy (SCRT) aims to reduce the overall treatment course (i.e., a reduction from ~five to six weeks down to ~one week) while maintaining oncological outcomes comparable to conventional chemoradiation [6]. Other investigators have noted encouraging rates of clinical complete response with SCRT for patients motivated for non-operative management [7,8]. The efficiency of treatment is also favored in patients with metastatic disease to mitigate patient logistical burden but also reduce time off systemic therapy during radiotherapy [9,10]. Furthermore, this strategy has been applied to an oligometastatic cohort (one-two metastatic organs, no peritoneal disease, only adenocarcinoma histology) treated to 25 Gy in five fractions to the rectal primary, and at a median follow-up of 16.6 months demonstrated a 17% rate of clinical complete response, and at one-year reported 89% local control and 83% overall survival [11].

To further these encouraging results, we hypothesized that isotoxic radiotherapy dose escalation may be best achieved with CT-based adaptive radiation therapy. This technique allows for daily modifications to the radiation plan based on inter-fractional anatomical changes during treatment as visualized with cone beam CT (CBCT) online imaging, thereby optimizing dose delivery and minimizing toxicity to surrounding tissues [12]. Other investigators have demonstrated the feasibility of this adaptive technique in treatment with the standard dose of 25 Gy in five fractions [13,14]. Additionally, a simultaneous integrated boost to 30 Gy in five fractions has been previously delivered without an adaptive platform, and with greater than two years of median follow-up, investigators did not identify excess anorectal toxicity [8].

This approach has yet to be demonstrated with an adaptive platform, which may provide the benefit of improving target coverage while maintaining bowel and bladder constraints, potentially reducing toxicity compared to conventional non-adaptive techniques. Additionally, the clinical efficacy of hypofractionation depends upon increased precision at each treatment fraction, as each individual treatment represents a larger portion of the total radiation dose to be delivered, and any inaccuracy could be more consequential than in standard-length radiotherapy courses. Online adaptation can help provide such daily precision. In this case report, we present a patient with metastatic rectal cancer who was treated with a non-operative approach with SCRT with moderate dose-escalation with a novel CT-based adaptive platform.

## Case presentation

A 42-year-old male presented to an outside institution with concerns of weight loss, diarrhea, and weakness. He was found to have significant anemia (Hgb 3.5) and elevated carcinoembryonic antigen (CEA) (>994); further workup with CT chest, abdomen, and pelvis demonstrated a large mass in the pelvis arising from the rectum with perirectal lymphadenopathy, numerous hepatic lesions, and few indeterminate sub-centimeter pulmonary nodules. Pelvic MRI showed this mass to be 14 cm in length and located 6-8 cm from the anal verge, straddling the anterior peritoneal reflection and invading the right seminal vesicle. These findings prompted a colonoscopy and the rectal mass was biopsied, finding pathology consistent with adenocarcinoma and microsatellite stable. Additionally, metastatic disease was confirmed on a liver biopsy. His initial clinical stage was mrT4bN2aM1b, stage IVB. He underwent diversion with colostomy and subsequently initiated systemic therapy with leucovorin calcium, 5-fluorouracil, oxaliplatin (FOLFOX), and bevacizumab.

**Figure 1:**
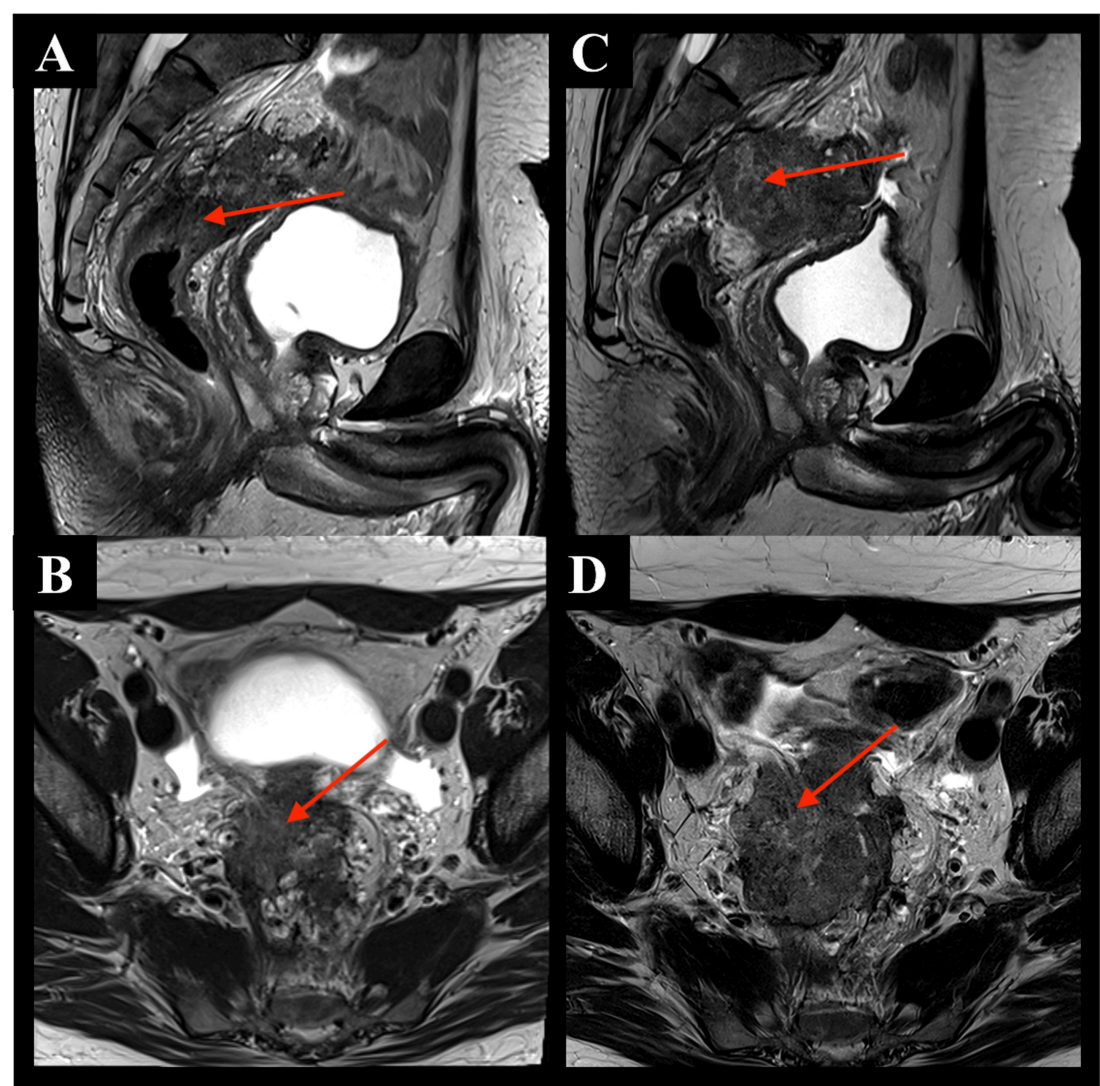
Restaging MRI after chemotherapy showing the rectal tumor 8.5 cm from the anal verge and 6.4 cm in craniocaudal length and pelvic MRI for radiation treatment planning demonstrating tumor progression, 6.8 cm in craniocaudal length. A: Restaging MRI sagittal view; B: Restaging MRI axial view; C: Radiation treatment planning MRI sagittal view; D: Radiation treatment planning MRI axial view. Red arrows indicate the primary tumor.

He sought a second opinion with medical oncology at our institution, and he was recommended to escalate treatment to fluorouracil, leucovorin, oxaliplatin, and irinotecan (mFOLFOXIRI) and bevacizumab. He ultimately received 14 cycles of this regimen with partial radiographic response in the liver and pelvis. Restaging pelvic MRI re-demonstrated a mid-high rectal tumor, 8.5 cm from the anal verge, 6.4 cm in craniocaudal length, with persistent invasion of the right seminal vesicle and enlarged mesorectal lymph nodes (Figures 1A, 1B). As he was relatively asymptomatic regarding his primary rectal disease, it was recommended that he hold systemic therapy and consider liver-directed therapy to establish hepatic disease control, followed by radiotherapy to the pelvis.

Two months post-bilobar transarterial radioembolization, he returned to the radiation oncology clinic, and he consented to pelvic short-course CT-based adaptive radiotherapy. The patient was educated to have a comfortably full bladder and empty rectum prior to treatment. Pelvic MRI for radiation treatment planning demonstrated primary tumor progression now 6.8 cm in craniocaudal length (Figures 1C, 1D).

**Figure 2:**
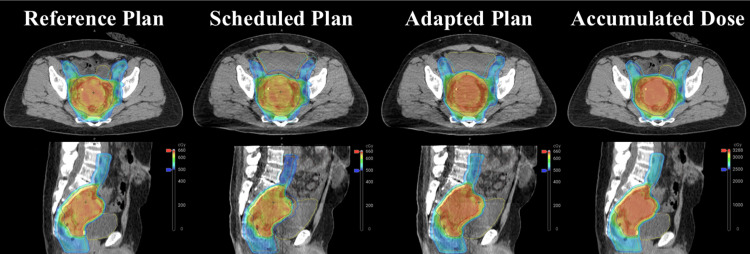
Visual comparison of the dosimetry in the reference plan (initial plan on simulation CT), scheduled plan (reference plan recalculated on image/anatomy of the day), and an adapted plan (plan fully reoptimized on image/anatomy of the day) for a representative treatment fraction. The variability in bladder (yellow) fill and subsequent change in bowel (brown), combined with a slight patient body habitus size increase, results in a superior adaptive plan to adequately cover the GTV (red), PTV high (dark blue), CTV low (orange), and PTV low (cyan) without exceeding dose tolerance of bladder or bowel. The accumulated dose approximated via deformable registration of each adapted fraction back onto reference CT and anatomy is also displayed. GTV: gross tumor volume; CTV: clinical target volume; PTV: planning target volume

The radiotherapy prescription was 25 Gy to the pelvis with coverage of the external iliac vessels and a simultaneous integrated boost to 30 Gy to the primary rectal tumor (Figure 2). This was delivered with a nine-field step-and-shoot intensity modulated radiation therapy (IMRT). Gross tumor volume (GTV) was contoured on the treatment planning CT with fusion of the treatment planning axial T2 weighted MRI performed at the time of CT simulation. The GTV was included in the pelvis clinical target volume (CTV), which was comprised of the mesorectum, presacral space, obturator, internal and external iliac lymph nodes, and common iliac lymph nodes. A 5 mm isotropic expansion was added to both the pelvic CTV and the GTV to generate the planning target volumes (PTV): PTV_2500 and PTV_3000, respectively. The dosimetric objectives included D95% of the PTV receiving prescription dose and D99% of the GTV and CTV receiving prescription dose (30 Gy and 25 Gy, respectively), overall plan D0.03cc ≤ 33 Gy. Dose-volume limits for the bladder included: D0.03cc ≤ 33 Gy, V25 Gy ≤ 15%, and for the bowel included: D0.03 cc ≤ 33 Gy, D65cc ≤ 25 Gy [8,15].

In terms of the adaptive radiotherapy workflow, the time from CBCT acquisition to treatment completion was 32.5 ± 4.1 (range: 28.4 - 37.7) minutes. This process includes initial CBCT acquisition, influencer structure generation and editing, target generation, remaining contour edits, plan generation, plan analysis and selection, verification CBCT, plan-specific QA, and treatment delivery. The influencer structures are defined by the treatment planner and are a subset of normal organ structures associated with the site being treated that are contoured via artificial intelligence and are assumed to deform similarly to other relevant organs and targets, such that the influencers are used to generate remaining structures of the day via structure-weighted deformable image registration.

For this treatment course, all five treatments were adapted per the decision of the physician primarily due to improved target coverage (Table 1). Adaptive re-planning with dose escalation also increased bowel D0.03cc on average by 3.2% across the treatment course from the scheduled plan delivery but still never exceeded the initial bowel constraint of D0.03cc < 33 Gy. The reason for the increase in bowel D0.03cc could be due to changes in anatomic geometry, as well as the effect of the optimizer to prioritize target coverage while keeping the organs at risk (OAR) doses within the prespecified ranges. As the Ethos optimizer strives to meet all high-priority clinical goals simultaneously, this may result in higher target coverage and an increased dose to an OAR while still adhering to the specified OAR constraints. Slight patient weight gain between simulation and treatments may also contribute to the observed changes. Changes in the bladder D0.03cc with adaptation were negligible (0.6%).

**Table 1:** Adaptive radiotherapy results comparing the dosimetric goals with the reference plan as well as the average achieved values for both scheduled and adaptive plans' overall treatment sessions. GTV: gross tumor volume; CTV: clinical target volume, PTV: planning target volume

Dosimetric Objective	Goal (per fraction)	Reference Plan	Scheduled Plan	Adapted Plan	Percent Difference (%)
Bladder, D0.03cc	660.0 cGy	657 cGy	647.6 cGy ± 8.8	651.6 cGy ± 2.3	0.6 ± 1.6
Bowel, D0.03cc	660.0 cGy	656 cGy	625.4 cGy ± 29.4	645.0 cGy ± 14.1	3.2 ± 3.1
GTV_3000, V30 Gy	99.00%	100%	99.3% ± 0.6	100.0% ± 0.0	0.7 ± 0.6
PTV_3000, V30 Gy	95.00%	95.60%	89.4% ± 2.9	96.7% ± 0.8	8.2 ± 3.9
CTV_2500, V25 Gy	99.00%	99.40%	97.7% ± 0.9	99.7% ± 0.3	2.1 ± 1.2
PTV_2500, V25 Gy	95.00%	97.50%	91.1% ± 1.9	97.8% ± 0.7	7.4 ± 2.8

The patient tolerated the treatment well with minimal fatigue and abdominal cramping during treatment, which self-resolved (Grade 1). Approximately one month following the completion of this treatment, he resumed 5-fluorouracil + bevacizumab and then escalated to FOLFOXIRI + bevacizumab due to the distant progression of the disease. He was seen for toxicity assessment with radiation oncology at two months post-SCRT and reported no new pelvic symptoms and resolution of his acute side effects from SCRT. 

## Discussion

Here, we present a case of a patient with metastatic rectal cancer treated with SCRT on a novel CT-based adaptive platform, which permitted moderate radiotherapy dose escalation for an inoperable patient. SCRT has increasingly been evaluated as a treatment option for locally advanced non-metastatic patients within the total neoadjuvant therapy paradigm. The recently published Rectal Cancer and Preoperative Induction Therapy Followed by Dedicated Operation (RAPIDO) trial enrolled high-risk patients in SCRT-based total neoadjuvant therapy (TNT) compared to the traditional standard of care of chemoradiation followed by surgery and consideration of adjuvant chemotherapy. The long-term results demonstrated that the TNT approach resulted in an improvement in the primary endpoint of disease-related treatment failure; however, an increased rate of locoregional failure was noted in the experimental arm, which drew attention to the SCRT technique and dose [16]. The phase II SHORT-FOX study (NCT04380337) is awaiting publication but seeks to evaluate the clinical complete response rate of SCRT dose escalation and includes chemotherapy intensification with FOLFOXIRI [17].

In the metastatic setting, SCRT is a more favorable radiotherapy schedule compared to chemoradiation to minimize time off combination systemic therapy. Additionally, the logistical considerations for patients with reduced life expectancy also favor a more expedient treatment course. However, the biologically effective dose (BED10) of traditional SCRT at 37.5 Gy is appreciably less than conventional chemoradiation techniques that reach a minimum BED10 of 59.5 Gy, which may have contributed to the results of the RAPIDO study. As there is evidence of a radiotherapy dose response in rectal cancer [18], methods to safely increase radiotherapy dose to gross disease should actively be explored. Adaptative radiotherapy may be a solution to increase the dose of the tumor while maintaining isotoxicity. In this case, by escalating the gross disease to 30 Gy in five fractions, the BED10 is increased to 48.0 Gy, potentially enhancing the efficacy of SCRT. Adaptive radiotherapy also provides the necessary daily precision to ensure doses to targets and normal tissue consistently meet clinical goals.

Averaged across the adaptive workflow for this patient, PTV coverage improved by 8.2% and 7.4% for the high and low dose volumes, respectively (Table 1). The percent change in the V3000 and V2500, respectively, between scheduled and adapted plans was calculated for each fraction. The average over these percent differences demonstrated that CT-based adaptive radiation improved high (30 Gy) and low dose (25 Gy) target coverage versus the scheduled plan on average by approximately 8% (ranges of 2.0-11.7% and 2.4-9.3%, respectively) while maintaining bowel and bladder constraints. Since adaptive radiation therapy involves adjustment to the radiation plan in response to changes in the patient’s anatomy or tumor size during the course of treatment, it may result in less toxicity when compared to traditional radiation therapy based on initial imaging.

De Jong et al. previously demonstrated the early feasibility of a CBCT-based adaptive radiotherapy workflow for rectal cancer [13]. All twelve patients from this study were compliant with the treatment regimen, the online adaptive workflow took an average of 26 minutes, and the adaptive plans achieved improvements in PTV coverage compared to scheduled plans [13]. In addition, Lutkenhaus et al. showed that adaptive plan selection based on daily CBCT scans in rectal cancer radiotherapy may incrementally reduce bladder and bowel dose for some patients compared to a non-adaptive approach, and this was predominately driven by a reduction in PTV expansions allowed by the adaptive platform [14]. In our experience, we noted similar findings in that dosimetric sparing of the bladder and bowel in our patient was limited; however, we found more dramatic improvements in target coverage. The representative value for CTV_2500 in Table 1 shows that the V25Gy was on average 2.1% better with adaptation compared to without. However, the goal of 99% CTV coverage by this dose was met in all adapted treatments but only in one of five scheduled plans. For the high dose GTV coverage metric (V30Gy), there was a negligible improvement (0.7%) in coverage, though the adapted plan met the 99% coverage goal in all fractions compared to the scheduled plan, which failed in one of five fractions. This may imply that the benefits of an adaptive workflow in rectal cancer are patient-dependent and location-specific (high/low rectum), and using a simultaneous integrated boost may further increase the advantage of this technique. 

However, there remains a need for further assessment of the clinical benefits of this approach more broadly in rectal cancer, as questions exist regarding the optimal patient selection for SCRT in patients with locally advanced disease [16]. Although our patient did not undergo surgery, the risks of postoperative complications after SCRT should be considered if this technique is broadened to a non-metastatic population. A recent meta-analysis from Yang et al. found that neoadjuvant radiotherapy may increase the risk of anastomotic leakage, wound infection, and pelvic abscess compared to upfront surgery in rectal cancer patients, with both short-course and long-course radiotherapy increasing the incidence of anastomotic leakage [19]. For colorectal surgery in general, surgical site infection is the leading postoperative complication following colorectal surgery, causing significant patient distress and is associated with increased healthcare costs, morbidity, and mortality [20,21]. With the increasing complexity in the management of rectal cancer, potential risks of trimodality therapy must be weighted to optimize the chance of cure while considering the patient’s functional outcome.

Future studies evaluating the clinical impact of CBCT-based online adaptive radiotherapy should use robust toxicity and patient quality-of-life assessments, as functional outcomes for patients with rectal cancer are of increasing importance. Improved target coverage and conformality with CT-based online adaptation suggest that future work could investigate how this improved precision in treatment delivery may allow for a reduction in PTV margins and further normal tissue sparing. A margin reduction strategy may provide additional OAR sparing while also accounting for daily contouring uncertainty. A similar margin reduction approach (down to 3 mm) is being evaluated in the context of CT-guided adaptive radiotherapy in the pelvis for cervical cancer [22,23]. To better determine what margins to use in the context of SCRT for rectal cancer, a larger sample size is warranted to examine the effects of the uncertainties associated with the online adaptive process. Coupled with robust quality-of-life measures, we anticipate that CT-based adaptive radiation has the potential to allow for selective radiotherapy dose escalation as we seek to improve clinical response rates.

## Conclusions

This case report illustrates the potential of CT-based adaptive radiation therapy in a patient with metastatic rectal cancer. Adaptive SCRT may help to mitigate toxicity while offering avenues to improve pelvic disease control or clinical complete response rates as a tolerable alternative to chemoradiation. Future prospective studies should also measure other clinical benefits, including toxicity reduction and assessment of patient quality of life.
